# Inpatient healthcare utilization among people with disabilities in Iran: determinants and inequality patterns

**DOI:** 10.1186/s12913-023-10383-0

**Published:** 2024-01-11

**Authors:** Badriyeh Karami, Satar Rezaei, Hadi Darvishi Gillan, Shahram Akbari, Raheleh Maleki, Fardin Moradi, Faramarz Jalili, Mohammad Karami, Shahin Soltani

**Affiliations:** 1 Research Center for Environmental Determinants of Health, Health Institute, Kermanshah University of Medical Sciences, Kermanshah, Iran; 2 School of Health Administration, Dalhousie University, Halifax, NS Canada; 3 Students Research Committee, Kermanshah University of Medical Sciences, Kermanshah, Iran

**Keywords:** Socioeconomic disparities, Concentration index, Hospitalization rates, Disability, Decomposition analysis

## Abstract

**Background:**

People with disabilities (PWD) have different health service needs and different factors affect the utilization of these services. Therefore, the aim of this present study was to identify determinants of inpatient healthcare utilization among PWDs in Iran.

**Methods:**

This research was a secondary data analysis of a cross-sectional study. The present study used data gathered for 766 PWDs (aged 18 years and older) within the Iranian Society with Disabilities (ISD) between September and December 2020. Multiple logistic regression models calculated adjusted odds ratios (aOR) and 95% confidence intervals in order to identify determinants of inpatient healthcare utilization among PWDs.

**Results:**

Data for 766 people with disabilities were analyzed. A large number of participants were over 28 years of age (70.94%), male (64.36%), and single (54.02%). In the present study, more than 71% of participants had no history of hospitalization during the last year. In this study, males [aOR 2.11(1.14–3.91), participants with Civil Servants health insurance coverage [aOR 3.44 (1.16 − 10.17)] and individuals in the 3th quartile of disability severity [aOR 2.13 (1.01 − 4.51)] had greater odds of inpatient healthcare utilization compared to the other groups. The value of the concentration index (C) for inpatient healthcare utilization was − 0.084 (*P*.value = 0.046). The decomposition analysis indicated that gender was the greatest contributor (21.92%) to the observed inequality in inpatient healthcare utilization among participants.

**Conclusion:**

Our findings suggested that the likelihood of hospitalization among the study participants could be significantly influenced by factors such as gender, the health insurance scheme, and the degree of disability severity. These results underscore the imperative for enhanced access to outpatient services, affordable insurance coverage, and reduced healthcare expenditures for this vulnerable population. Addressing these issues has the potential to mitigate the burden of hospitalization and promote better health outcomes for disadvantaged individuals.

## Introduction

People have the basic right to health, which contribute to people’s preference of an acceptable level of overall health, financial protection against high healthcare costs, and responsiveness to the clinical and non-clinical needs of people who seek health services; these are the main goals of the health system [1]. International policy documents, including the Declaration of Alma-Ata [2], the 2030 Agenda for Sustainable Development [3], and the United Nations Convention on the Rights of Persons with Disabilities (UNCRPD) [4] have referred to this issue.

The World Health Organization (WHO) estimates that more than one billion people, or 15% of the world’s population, live with a disability, 80% of which are in low- and middle-income countries [5]. Based on the annual survey of Eurostat statistics on income and living conditions in Europe, the prevalence of disability is higher among female, older and less educated [6].

To support the people with disabilities, there are various policies in the world. The World Health Assembly has emphasized health systems policies and research on disability through the resolution. “Resolution on the Highest Attainable Standard of Health for Persons with Disabilities” which member states requested WHO “to establish a support the global research program. which aligns with UHC, health emergencies and health and well-being, including health systems and policy research” [7].

Also, in Iran, according to the law on the protection of the rights of people with disabilities of the Islamic Council, the Ministry of Health, Treatment and Medical Education is obliged to provide health insurance coverage for people with disabilities covered by the organization in such a way that in addition to providing the medical services needed by these people, physical rehabilitation services and cover the mental health of people with disabilities [8].

Despite these laws, PWDs often experience a lower level of health than healthy people for various reasons [9]. PWDs disproportionately experience unmet healthcare needs and health disparities [10]. According to international evidence, PWDs around the world face certain barriers when accessing healthcare services [11, 12].

In general, PWDs have poorer access to health care despite a greater need for them [13, 14], and inequality in access to health care is a global issue that leads to poorer health outcomes [15]. In addition, this inequality is considerable among PWDs. A recent survey by the WHO shows that 76–85% of PWDs living in developing countries do not receive any healthcare and only 2–3% of these people can access rehabilitation services [16]. Various factors affect access to health services. The results of the study by Shamyr (2011) [17] showed that people with multiple or mobility disabilities and who are less than 78 years of age, and people who experience transportation barriers to health services centers, were more exposed to health service access problems. Therefore, knowing these factors which affect the health service utilization can be an important health indicator to use in improving health care services and utilization for people with disabilities.

In various studies [18, 19], the socio-economic factors affecting patients health services utilization have been examined. The study of Asghari et al. [20] has investigated health services utilization in mentally disabled children and the factors affecting them, but no study has been conducted that examines the socioeconomic factors affecting the hospitalization rates of people with different degrees of disabilities. Therefore, the main objectives of the present study included: To identify determinants of inpatient healthcare utilization; To measure socio-economic inequalities in inpatient healthcare utilization; and to determine major contributors to the socioeconomic-related inequality in inpatient healthcare utilization among PWDs in Iran.

## Methods

### Study design

Our study was a secondary data analysis of a cross-sectional dataset.

### The study participation

In the present study data for 786 PWDs were analyzed. PWDs were recruited from the Iranian Society with Disabilities (ISD) between September and December 2020. The ISD is a non-governmental organization (NGO) that facilitates access to education and healthcare services for PWDs in all provinces of Iran [21]. At the time of the study, about 50,000 people with disabilities ≥ 18 years were member of the ISD. This institution has 19 branches and 47 local offices nationwide and provides various social, financial, educational and health support to its members. The sampling method in this study was convenience sampling. People with disabilities aged 18 and over and members of ISD with Iranian nationality, were included in the study.

### Data collection

The sample of this study was 786 individuals. The sampling method used in this study was the convenience sampling method. To measure socioeconomic inequality, Karami Matin et al.‘s valid and reliable questionnaire was used [22]. This self-reported questionnaire contains questions related to demographic characteristics, socioeconomic status, and access to rehabilitation services. Additionally, the Washington Group short questionnaire on functioning was used to determine the type and the severity of disability [23]. Questions have been developed according to the most basic level of functioning: hearing, seeing, walking or climbing steps; remembering or concentrating; selfcare, and communicating. The six functions were adopted as universal, happening generally, and related to social inclusion. Also, the severity of functional limitation in each item was assessed by a four-point scale: “no difficulty”, “some difficulty”, “a lot of difficulties”, and “unable to do it”. Further details about this questionnaire have been reported by Palmer and Harley [24].

Due to the COVID-19 pandemic, an electronic format was used in collecting the data, and the link of the electronic questionnaire was sent to ISD members through messaging applications i.e., Telegram and WhatsApp.

### Variables

The outcome variable was a binary variable showing whether the participants were hospitalized in a hospital during last year.

In this study, the severity of functional limitations was examined by a four-point scale: “no difficulty”, “some difficulty”, “a lot of difficulty”, and “unable to do it”. For further details on this questionnaire see the study by Palmer and Harley [25].

In this study, we utilized data on asset ownership (such as cars, microwave oven, twin refrigerators/side by side refrigerator, vacuum machine, personal computer, washing machine, and dishwasher) and housing features (such as private or rental housing and house area), and educational level of the participants to develop SES variable based on existing data.

To develop SES indicator, we employed Filmer and Pritchett’s method [26], which utilizes principal components analysis (PCA) to reduce multidimensional datasets on household asset ownership to a lower number of dimensions. This approach allowed us to divide the participants into five wealth quintiles, ranging from the lowest (1st quintile) to the highest (5th quintile) groups.

In our study, we examined several potential contributors to the observed socioeconomic inequality in poor financial access to rehabilitation services. These contributors included:

- Gender: Participants were categorized as male or female; Age: Age groups were defined as 18–27 years, 28–37 years, 38–47 years, 48–57 years, and 58 years or older; Marital Status: Participants were classified as single, married, or widowed/divorced; Place of Residence: Participants were categorized based on their place of residence (urban or rural areas); Head of Household: Participants were identified as either the head of the household or not; Health Insurance coverage: Health insurance coverage was categorized into groups such as no insurance, Social Security, Military, Universal Health Insurance, Civil Servants, and Other; Education: Education levels were classified as illiterate, primary school, secondary school, high school, and academic.

### Data analysis

Firstly, the multiple logistic regression model was applied to calculate adjusted odds ratios (aOR) and 95% confidence intervals in order to identify determinants of inpatient healthcare utilization among PWDs.

Also, we used the concentration index (C) to measure the socio-economic inequalities in inpatient healthcare utilization among participants. The C is a standard measure to quantify income-related inequalities in health economics [27]. This index is twice the area between the concentration curve and the 45° line indicating no relationship between the two variables [28]. The concentration curve serves as the bivariate counterpart to the Lorenz curve, providing a visual representation of the cumulative proportion of hospitalizations relative to the cumulative proportion of the population, categorized by their socio-economic status (SES). In situations where income-related inequalities do not exist, the concentration index attains a value of zero.

As per convention, the concentration index assumes a negative value when the curve extends above the line of equality. This signifies an imbalanced concentration of the health variable among individuals with lower socio-economic status, often denoting economic disadvantage. Conversely, a positive value is assigned to the concentration index when the curve falls below the line of equality. Concerning health variables, especially those categorized as ‘undesirable,‘ such as poor health, a negative concentration index suggests a higher prevalence of the health issue within the economically disadvantaged population. The Concentration index is defined as [29]:

$$C\left(h\vert y\right)=\frac{2\;cov\left(h_i,\;R_i\right)}{\overline h}=\frac1n\sum\limits_{n=1}^n\left[\frac{h_i}{\overline h}\left(2R_i-1\right)\right]$$

The C ranges from 1 − n/n (maximal *pro-poor* inequality i.e. the health outcome is concentrated on the poorest individual) to n − 1/n (maximal *pro-rich* inequality).

In addition, C was decomposed to measure the proportion of different socio-economic variables in inequalities.

The estimated value of the normalized C was decomposed to identify the contribution of explanatory variables to the observed socioeconomic inequality in inpatient healthcare utilization. Wagstaff and colleagues [30] showed that if we have a regression model relating a health outcome variable of $y$ to a set of $k$ explanatory variables, $x,$ such as:

1 $$y=\alpha +\sum _{k}{\beta }_{k}\,{x}_{k}+ \varepsilon,$$

The C for $y$ can be decomposed as:

2 $$C=\sum_{k}\left( \frac{{\beta }_{k}{\stackrel{-}{x}}_{k}}{\mu }\right){C}_{k}+G{C}_{\varepsilon}/\mu.$$

In this equation, ${\stackrel{-}{x}}_{k}$ indicates the mean of the explanatory variable, $x$, ${C}_{k}$ is the C for each explanatory variable, and $G{C}_{\varepsilon }$ shows the generalized C for $\varepsilon$. In Eq. 2, the first component $\sum _{k}\left( \frac{{\beta }_{k}{\stackrel{-}{x}}_{k}}{\mu }\right){C}_{k}$ shows the contribution of explanatory variable $x$ to the overall socioeconomic-related inequality in the outcome variable. The positive contribution of an explanatory variable explains that the SES-related distribution of this variable increases the concentration of inpatient healthcare utlization among the high SES individuals. Also, in Eq. 2, the second component, $\frac{G{C}_{\varepsilon }}{\mu }$ indicates the proportion of socioeconomic inequality in inpatient healthcare utilization which is not explained by the systematic variation of the included explanatory variables across SES groups. Applying Wagstaff’s [31] correction into Equation results in:

3 $${C}_{n}=\frac{C}{1-\mu }=\frac{\sum _{k}\left(\frac{{\beta }_{k}{\stackrel{-}{x}}_{k}}{\mu }\right){C}_{k}}{1-\mu }+\frac{G{C}_{\varepsilon }/\mu }{1-\mu }$$

Given that inpatient healthcare utilization was a binary variable, we used marginal effects derived from a logistic model as $\beta$ in the decomposition of the ${C}_{n}$. Stata version 14.2 software (Stata Corp, College Station, TX, USA) was applied for data analysis

## Results

### Characteristics of participants

Following the exclusion of missing data, a total of 766 individuals with disabilities were included in the data analysis. A large number of participants were over 28 years of age (70.94%), the majority of the sample were male (64.36%), and single (54.02%), and persons living in urban area (88.33%). Most of respondents (72.39%) are in the first quartile in terms of severity of disability. In terms of socioeconomic status, they are almost equally distributed in the quintiles (Table 1).

**Table 1:** Summary characteristics of the study population

Variables	Study population (%)
Sex	
Male	493 (64.36)
Female	273 (35.64)
Total	766
Missing value	20
Age groups (years)	
18–27	69 (10.66)
28–37	218 (33.69)
38–47	241 (37.25)
48–57	98(15.15)
>=58	21 (3.25)
Total	647
Missing value	124
Marital status	
Single	403 (54.02)
Married	301 (40.35)
Widowed and divorced	42 (5.63)
Total	746
Missing value	40
Place of residence	
Urban setting	681 (88.33)
Rural setting	90 (11.67)
Total	771
Missing value	15
Head of households	
No	418 (54.71)
Yes	346 (45.29)
Total	764
Missing value	22
Disability severity	
1^st^ quartile (low severity)	259 (39.72)
2^nd^ quintile	125 (19.17)
3^rd^ quintile	165 (25.31)
4^th^ quartile (high severity)	103 (15.80)
Total	652
Missing value	134
Insurance	
No insurance	83 (10.91)
Social Security	331 (43.50)
Military	21 (2.76)
Universal Health Insurance	189 (24.84)
Civil Servants	91 (11.96)
Other	46 (6.04)
Total	761
Missing value	25
SES	
1^st^ quintile (the lowest)	120 (20.13)
2^nd^ quintile	120 (20.13)
3^rd^ quintile	118 (19.80)
4^th^ quintile	120 (20.13)
5^th^ quintile (the highest)	118 (19.80)
Total	596
Missing value	170

### Hospitalization frequency among people with disabilities

**Fig. 1:**
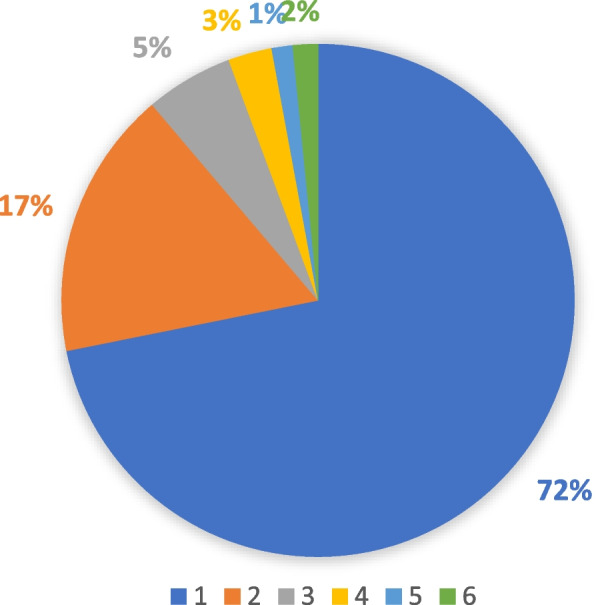
The rate of Hospitalization frequency

According to Fig. 1 and 71.84% of participants had no history of hospitalization during the last year. About 17% of participants used inpatient healthcare once a year, while others were hospitalized twice or more than two time within a year.

### Determinants of relationship between hospitalization rate and demographic and socioeconomics characteristics of participants

As shown in Table 2, men were 2.11 times more likely to use inpatient healthcare compared to women. The Civil Servants health insurance scheme was associated with a higher hospitalization rate [aOR 3.44 (1.16 − 10.17)]. Also, individuals in the 3th quartile of disability severity had 2.13 times greater odds [ aOR 3.44 (1.01 − 4.51)] of hospitalization compared to the 1st quartile.

**Table 2:** Odds ratios of the occurrence of hospitalization among the survey respondents (*N* = 786)

Hospitalization	Odds Ratio	Std. Err.	*P* value	[95% Conf. Interval]	
**Gender (ref: female)**	2.114	0.664	0.017	1.142	3.913
**Age groups (ref: 18–27 years)**					
28–37	1.490	0.727	0.413	0.573	3.875
38–47	0.948	0.493	0.918	0.342	2.627
48–57	0.596	0.380	0.416	0.171	2.079
>=58	2.937	2.808	0.260	0.451	19.126
**Marital status (ref: married)**	1.129	0.356	0.699	0.609	2.094
**Place of residence (ref: urban setting)**	1.230	0.502	0.611	0.553	2.737
**Health insurance (ref: no insurance)**					
Social Security	1.815	0.792	0.172	0.772	4.267
Military	3.677	2.907	0.100	0.781	17.317
Universal Health Insurance	0.755	0.357	0.551	0.299	1.905
Civil Servants	3.443	1.903	0.025	1.165	10.175
Other	1.507	0.987	0.531	0.417	5.442
**Disability severity (ref: 1**^**st**^ **quartile)**					
2^nd^ quartile	1.542	0.527	0.205	0.790	3.013
3^rd^ quartile	2.131	0.817	0.048	1.006	4.517
4^th^ quartile (high severity)	1.155	0.402	0.679	0.584	2.284
**Education (ref: illiterate)**					
Elementary	0.986	1.067	0.990	0.118	8.214
Secondary	0.764	0.772	0.790	0.105	5.547
High school	2.297	2.175	0.380	0.359	14.698
Academic	1.507	1.390	0.657	0.247	9.184
**Wealth (ref: 1**^**st**^ **quintile)**					
2^nd^ quintile	1.283	0.519	0.538	0.581	2.834
3^rd^ quintile	2.154	0.857	0.054	0.987	4.699
4^th^ quintile	0.655	0.277	0.316	0.286	1.499
5^th^ quintile (the highest)	0.385	0.175	0.036	0.158	0.940

### Socioeconomic inequality in inpatient healthcare utilization

**Fig. 2:**
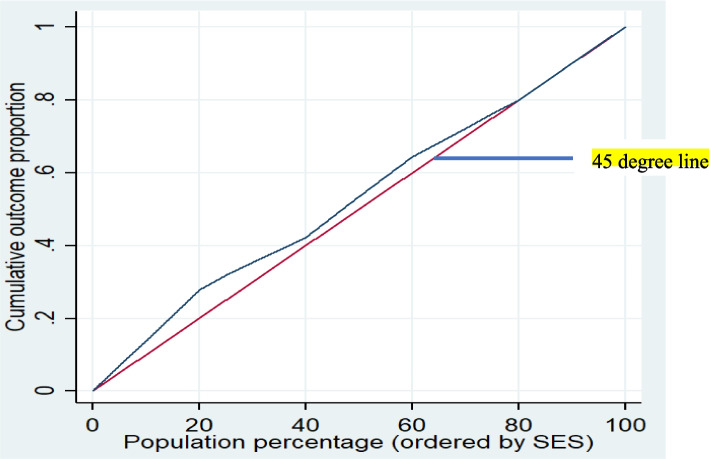
The Lorenz graph shows the cumulative distribution of inpatient healthcare services over the cumulative population ordered by SES

As shown in Fig. 2, in this study the value of the concentration index (C) for the occurrence of hospitalization was − 0.084 (*p*-value = 0.046, Standard error = 0.073), indicating that the occurrence of hospitalization was concentrated among the participants with lower SES.

**Fig. 3:**
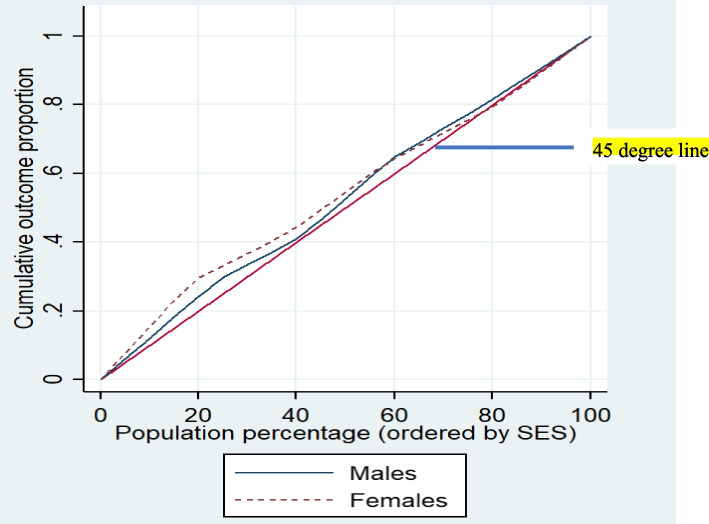
The Lorenz graph shows the cumulative distribution of inpatient healthcare services over the cumulative population ordered by SES among men and women with disparities

In addition, the value of the concentration index (C) for the occurrence of hospitalization for men and women were − 0.077 (*p*-value = 0.442, Standard error = 0.1), and − 0.095 (*p*-value = 0.038, Standard error = 0.11), respectively, indicating that the occurrence of hospitalization was concentrated among women with lower SES than men (Fig. 3).

### Decomposition analysis of socioeconomic-related inequalities in inpatient healthcare utilization

Regarding Table 3, the decomposition analysis indicated that gender was the greatest contributor (21.92%) to the observed inequality in inpatient healthcare utilization among participants. In other words, decomposition analysis reveals that being male is associated with lower SES and higher likelihood of inpatient healthcare utilization. This positive contribution is a result of both the negative C for males and the positive elasticity of all measures of inpatient healthcare utilization with respect to gender status.

**Table 3:** Decomposition analysis of socioeconomic-related inequalities in inpatient healthcare utilization

Variables	Partial effects	Mean	Elasticity	Concentration Index (C_k_)	Absolute Contribution	Percentage contribution	Summed Percentage Contribution
**Gender**							
Female							
Male	0.129	0.627	0.288	-0.046	-0.018	21.923	21.92
**Age**							
18–27							
28–37	0.061	0.277	0.060	-0.053	-0.004	5.277	4.97
38–47	-0.033	0.306	-0.036	-0.022	0.001	-1.309	
48–57	-0.141	0.124	-0.062	0.044	-0.004	4.533	
>=58	-0.164	0.025	-0.015	-0.146	0.003	-3.527	
**Marital status**							
Married							
Single	-0.035	0.512	-0.064	-0.033	0.003	-3.484	-3.03
Others (widow, divorced)	0.047	0.053	0.009	-0.031	0.000	0.455	
**Education**							
Illiterate							-28.80
Primary school	-0.016	0.067	-0.004	-0.259	0.001	-1.636	
Secondary school	-0.031	0.143	-0.016	-0.213	0.005	-5.564	
High school	0.120	0.207	0.088	-0.021	-0.003	3.074	
Academic	0.065	0.554	0.128	0.128	0.023	-27.159	
**Head of household**							
No							
Yes	-0.008	0.442	-0.013	0.000	0.000	0.000	0.00
**Place of residence**							
Urban setting							
Rural setting	0.029	0.114	0.012	-0.457	-0.007	8.902	8.90
**Disability severity**							
Mild							
Moderate	0.074	0.191	0.050	-0.059	-0.004	4.914	7.053
Severe	0.150	0.154	0.082	-0.010	-0.001	1.361	
Profound	0.043	0.256	0.039	-0.012	-0.001	0.778	
**Health insurance**							
No insurance							
Social Security	0.130	0.434	0.201	0.025	0.007	-8.311	-64.16
Military	0.158	0.027	0.015	0.346	0.007	-8.697	
Universal Health Insurance	0.010	0.248	0.009	-0.198	-0.002	2.893	
Civil Servants	0.181	0.119	0.077	0.407	0.043	-51.654	
Other	0.057	0.060	0.012	-0.080	-0.001	1.612	
**Wealth index**							
1^st^ quintile (the lowest)							
2^nd^ quintile	0.049	0.194	0.034	-0.302	-0.014	16.916	-36.76
3^rd^ quintile	0.141	0.201	0.101	-0.083	-0.012	13.860	
4^th^ quintile	-0.006	0.203	-0.004	0.175	-0.001	1.256	
5^th^ quintile (the highest)	0.096	0.201	0.069	0.605	0.058	-68.787	
**Explained**					0.08		-89.88
**Residuals**					0.17		189.88
**Total**					0.25		100.00

## Discussion

This study aimed to identify the determinants of hospitalization rate among people with disabilities in Iran. Based on the findings, more than 28% of participants had at least one hospitalization in the last year and occurrence of hospitalization was concentrated among the participants with a lower SES.

In our study, C was negative, which indicates a high level of hospitalization rate in people with lower socioeconomic status, which has been confirmed in previous studies [18]. Thomas and Ellis [32], in the United States, found that among Medicaid enrollees with disabilities who utilize outpatient services, the extent of service utilization is inversely linked to employment rates. In other words, individuals who use these services most frequently, specifically those with 54 or more days of usage, had the lowest employment rates. Conversely, they observed that there is a positive correlation between the diversity of service utilization and employment, indicating that individuals with higher socioeconomic status are more likely to access a wide range of healthcare services.

In the study by Ahmadi et al. [33], in Iran, the use of specialized medical and dental services, inequality was in favor of high-income groups, and for general medical, family doctor, and primary health care services, inequality was in favor of low income groups. Additionally, Nooraei Motlagh [34], and Lorent [35] showed that economic status has the largest contribution to inequality in the use of outpatient and inpatient health services. Various studies [36–40] indicate that people with a higher SES and those who have health insurance coverage are more likely to receive outpatient services than inpatient care.

Studies highlighted that PWDs due to disability and associated health problems need more inpatient care compared to people without disabilities. For example, Venkata et al. indicated that PWDs (18.4%) significantly needed to visit a hospital more often during a year compared to people without a disability (8.8%) [41]. Also, studies indicate that SES can affect unmet need for disability-related health care services among adults with disabilities. Accordingly, Henry et al. [42], in the USA, reported that PWDs with greater unmet healthcare needs were significantly less likely to be working. Their unmet needs were particularly greater for physical health services (durable medical equipment, personal assistance services, supplies) and medications.

Our findings showed that men were more likely to use more inpatient healthcare compared to women. Also, decomposition analysis showed that being male is associated with lower SES and higher likelihood of inpatient healthcare utilization. This result was due to negative C_k_ for males and the positive elasticity for all measures of inpatient healthcare utilization with respect to the male gender. There can be several factors such as different healthcare–seeking behaviors, type and severity of disability, socioeconomic factors, communication, support systems, health literacy, and geographic and regional differences that contribute to the observation [43–50]. For example, Kung et al. [51], in Taiwan, reported that the rate of preventive health services is significantly higher among women with disabilities compared to men. Some studies note that women demonstrate a heightened awareness of their well-being in contrast to men, resulting in a higher utilization of preventive or/and outpatient services by the female population [52, 53].

Decomposition analysis indicated that health insurance has made a negative contribution to socioeconomic-related inequality in inpatient healthcare utilization. A negative C_k_ indicates that this factor has a negative impact on reducing the observed inequalities. In contrast, positive elasticity suggests that the utilization of inpatient healthcare services is positively responsive to the health insurance scheme. In other words, the presence of this scheme is associated with an increase in healthcare utilization. Overall, the result suggests that, among participants with disabilities, the health insurance scheme is having a positive impact on inpatient healthcare utilization. However, despite this positive effect, the overall socioeconomic-related inequalities in healthcare utilization still exist, and this may be due to other negative factors (such as negative C_k_ coefficients) offsetting the benefits of the health insurance scheme.

The study by Yahyavi Dizaj et al. [55] shows that the use of rehabilitation services was higher in households ranked in higher income groups. Regarding the incomplete insurance coverage for rehabilitation services, people with a higher SES have more chances to use such services which in turn can reduce the probability of the occurrence of hospitalization among PWDs. Also, it should be noted that people with severe disabilities may need to receive appropriate home care services, but these services are not covered by health insurances in Iran which can increase the possibility of hospitalization among PWDs in low-income groups.

Also, the results of logistic regression analysis indicate that people with civil servant insurance 3.44 were more likely to be hospitalized than people without insurance coverage. Regarding a direct relationship between insurance coverage and the quantity of service utilization [54, 55], this group can use more of these services compared to uninsured people. It can also be said that coverage of home care and rehabilitation services by military insurance is better than other insurances, so the inpatient health services need is reduced. Additionally, the results of Tajvar et al. [54] indicate that people who did not have basic insurance access were less likely to receive outpatient care compared to those without basic insurance, and this factor can lead to increase in hospitalization rates .

A negative summed percentage contribution for the wealth index indicates that, in the context of the decomposition analysis, the wealth index has a negative impact on these inequalities. In other words, higher wealth is associated with reduced inpatient healthcare utilization among individuals with disabilities, and this effect is strong enough to result in a negative overall contribution when considering all the factors involved in the analysis. The negative value of this parameter suggests that the wealth index’s contribution to inequalities is in the opposite direction of reducing healthcare utilization. In the context of healthcare access and utilization, a negative contribution from wealth indicates that higher wealth is associated with a lower likelihood of utilizing inpatient healthcare services among individuals with disabilities. This could be due to a variety of factors, such as financial barriers, access to alternative healthcare options, or differences in healthcare-seeking behaviors among wealthier individuals with disabilities [56–58].

Furthermore, decomposition analysis revealed that education has made a negative contribution to the observed inequality in inpatient healthcare utilization. The results of this analysis suggest that among participants with disabilities, higher education levels (such as academic level) are associated with increased inpatient healthcare utilization. However, paradoxically, this increased utilization might contribute to negative socioeconomic-related inequalities, meaning that individuals with higher education levels tend to be poorer and use more inpatient services. Understanding these dynamics is essential for addressing healthcare inequalities and tailoring interventions to ensure equitable access and utilization of healthcare services.

### Limitations

Firstly, the emergence of the COVID-19 pandemic in Iran posed significant impediments to access PWDs, thereby exerting an impact on the data collection process. Furthermore, the utilization of online data collection methods may have inadvertently excluded individuals with disabilities who lacked access to essential communication tools, such as mobile phones or computers, as well as those with limited educational attainment. Consequently, it is imperative to acknowledge that our sample may not offer a comprehensive representation of the entire PWD population.

Also, the prevalence of the Covid-19 pandemic during the present study might have influenced the accessibility of inpatient healthcare services and hospitalization rates among PWDs. Additionally, it is worth noting that the exclusion of participants under the age of 18 in this study could potentially impact the generalizability of the results. The cross-sectional nature of this study, the lack of accurate clinical information about the disability status of participants, and self-reporting measures were the other limitations of this study.

## Conclusion

Our findings suggested that the likelihood of hospitalization among the study participants could be significantly influenced by factors such as gender, the health insurance scheme, and the degree of disability severity. These results underscore the imperative for enhanced access to outpatient services, affordable insurance coverage, and reduced healthcare expenditures for this vulnerable population. Addressing these issues has the potential to mitigate the burden of hospitalization and promote better health outcomes for disadvantaged individuals.

## Data Availability

Data and all other materials for this study are kept at the deputy of research and technology of Kermanshah University of Medical Sciences. The datasets generated and/or analyzed during the current study are not publicly available due to the terms of consent in which the participants agreed too, but which are available from the corresponding author on reasonable request.

## Abbreviations

PWD: People with disabilities
C: Concentration Index
SES: Socioeconomic Status
PCA: Principal Component Analysis
ISD: Iranian Society with Disabilities (ISD)
